# Supplementary material for: Assessment of Preferences in Taking Painkillers among Students of Medicine, Dentistry, and Pharmacy: A Pilot Study
Source: Healthcare (Basel). 2024 Jan 13;12(2):196. doi: 10.3390/healthcare12020196 (PMC10815322; doi:10.3390/healthcare12020196)
Supplement: Supplementary file 1 [file healthcare-12-00196-s001.zip › healthcare-2798320-supplementary.pdf]

**Assessment of the preferences in the taking of painkillers among students of medicine, dentistry, and pharmacy**

1. Gender:
    a. Female
    b. Male
2. Field of study:
    a. medicine
    b. dentistry
    c. pharmacy
3. Year of the study:
    a. 1st
    b. 2nd
    c. 3rd
    d. 4th
    e. 5th
    f. 6th
4. How often do you use painkillers?
    a. Less than once per month
    b. Once a month
    c. Once a week
    d. More than once a week
5. Pain threshold at which you use a painkiller:?
    a. Very severe/unbearable pain
    b. Severe pain
    c. Medium/moderate pain
    d. Mild pain
    e. To prevent expected pain
6. Do you comply with the recommended doses of a drug?
    a. yes
    b. rather yes
    c. rather no
    d. no
7. What do you consider most important when choosing a painkiller?
    a. fast onset of action
    b. strength of analgesic effect
    c. no side effects expected
    d. long duration of analgesic effect
    e. drug availability
    f. price
8. Do you have contraindications to the use of painkillers?
    a. Yes
    b. No
9. Are you concerned about side effects when using mild painkillers?
    a. Yes
    b. No
10. What drug do you choose for acute pain?
    a. ibuprofen
    b. acetaminophen

    c. metamizole
    d. acetaminophen combined with opioid
    e. aspirin
11. Do you consider advertisements when choosing painkillers?
    a. No, never
    b. Yes, sometimes
    c. Yes, often
    d. Yes, always
12. Where do you get information about individual painkillers from?
    a. studies curriculum
    b. a drug leaflet
    c. doctors and pharmacists
    d. the Internet
    e. friends and family
13. What is the most common side effect of painkillers?
    a. gastrointestinal complaints
    b. headaches and dizziness
    c. urticaria
    d. anaphylactic shock
    e. excessive sweating
14. Have you experienced toothache?
    a. Yes
    b. No
15. What over-the-counter drug would you recommend for toothache?
    a. Ibuprofen
    b. Acetaminophen
    c. No, A prescription drug would be my first choice
    d. I would not recommend any
    e. Metamizole
    f. Aspirin
16. What prescription drug would you recommend for toothache?
    a. Ketoprofen
    b. Nimesulide
    c. Tramadol
    d. I wouldn't choose any prescription drug
    e. Celecoxib
17. What is most important in combating dental pain with an analgesic?
    a. duration of action
    b. fast onset of action
    c. no or insignificant side effects
    d. simultaneous anti-inflammatory effect
    e. therapeutic safety
    f. convenient drug presentation
    g. Other
